# Maggot Debridement Therapy of a Leg Wound From Kaposi's Sarcoma: A Case Report

**DOI:** 10.1200/JGO.2015.001594

**Published:** 2015-11-25

**Authors:** Yuankai Lin, Molly Amin, Abigail F.W. Donnelly, Surabhi Amar

**Affiliations:** Yuankai Lin, Molly Amin, Xuan Nguyen, and Surabhi Amar, Maricopa Medical Center, Phoenix; Abigail F.W. Donnelly, Mayo Clinic, Scottsdale, AZ; and Surabhi Amar, University of Arizona College of Medicine-Phoenix, Phoenix, AZ

## INTRODUCTION

Malodorous, fungating wounds in the final stages of life for a patient with cancer have remained a challenge to physicians and wound care nurses as a result of the incurable nature of the disease and the compromised immunity of the host. Possible approaches to this problem include surgical debridement, vacuum-assisted closure, systemic antibiotics, and odor-controlling and medicated dressings; however, the outcomes are often unsatisfactory, and sometimes sharp wound debridement and vacuum-assisted closure are contraindicated for fungating wounds because of the risk for potential bleeding and malignant cell seeding.

The beneficial effect of maggot debridement therapy (MDT) on wound debridement has been known to surgeons since 1557.^1^ Baer popularized this method as early as 1931 when he reported the successful treatment of 89 cases of intractable osteomyelitis by the application of blow fly larvae,^2^ but its popularity soon waned with the introduction of antibiotics and antiseptic solutions. In the last 30 years, with the rising incidence of drug resistance, there has been renewed interest in using maggots in chronic wound therapy. Many cases in literature have reported successful application of MDT in a variety of wounds, including pressure ulcers, diabetic foot ulcers, venous ulcers, necrotizing fasciitis, crush injuries, burn wounds, wound infection after breast surgery, and so on. The average success rate is approximately 70%.^3^ A meta-analysis of 12 small clinical trials confirmed that MDT not only shortened the healing time but also improved the healing rate of chronic ulcers.^4^

Several attempts to debride different malignancy wounds by spontaneous myiasis or artificial MDT have also been made with various outcomes. Successful debridement was reported in necrotic wounds from cancer of the head and neck,^5–7^ squamous cell carcinoma of upper chest,^8^ and localized foot metastasis from endometrial adenocarcinoma.^9^ There is also a report of a failed attempt to treat a nonhealing lymph node biopsy site in a patient with mantle-cell lymphoma by using MDT.^10^ In this case, the failure was attributed to excessive exudate of lymphatic material from the wound. We report what is to our knowledge the first case of maggot therapy in a Kaposi sarcoma–related leg wound that saved the patient from a lower extremity amputation. The literature following the case report reflects the current understanding of MDT in cancer wounds, including its mechanisms and potential complications.

## CASE REPORT

**Figure 1:**
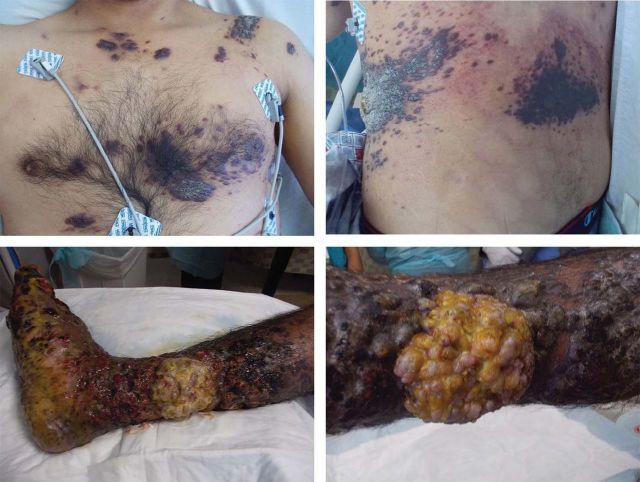
Severe skin lesions on torso and right lower extremity before the treatment.

The patient was a 26-year-old Hispanic male diagnosed with AIDS who was being treated with a combination of oral tenofovir plus emtricitabine as well as lopinavir plus ritonavir in Mexico. Despite 2 months of highly active antiretroviral therapy, he continued to have a severely depressed CD4 cell count of six cells per microliter and an HIV viral load of 10,700 copies per milliliter. He then moved to the United States and presented at our hospital. He also reported purplish, hyperkeratotic lesions all over his body (Fig 1) over the previous 3 to 4 months. These lesions encompassed nearly 50% of his body-surface area. The worst lesion was involving his right foot and lower leg, which was a large 10 × 10-cm fungating purulent mass on the medial aspect of his right calf (Fig 1). Along with the lesions, the ipsilateral foot had severe edema and dry gangrene. Surgical debridement was considered but not initiated because of fear that it would lead to an above-the-knee amputation, given a lack of viable tissue for closure.

**Figure 2:**
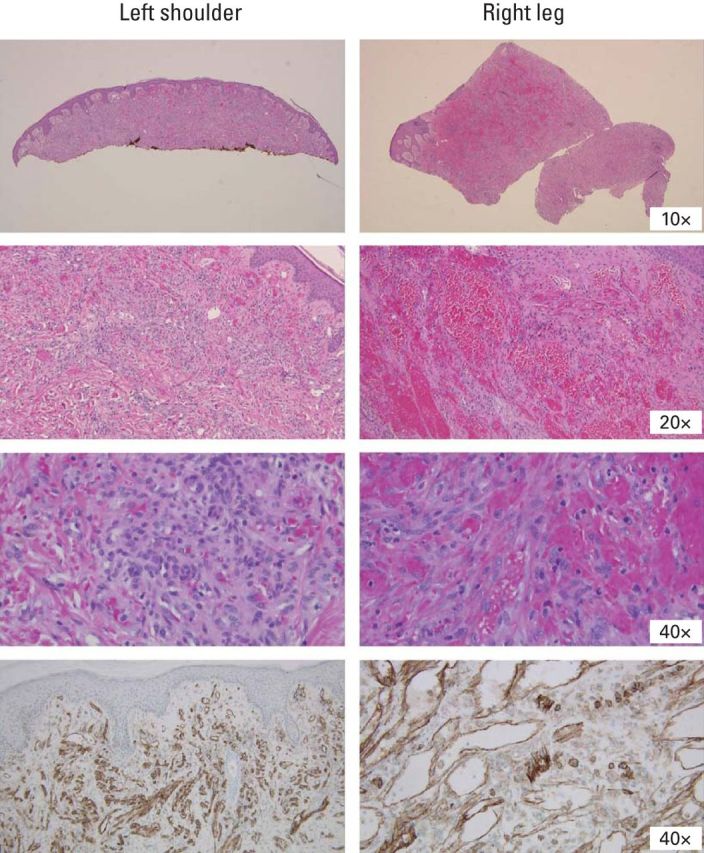
Punch biopsy from left shoulder and right leg confirmed the diagnosis of Kaposi's sarcoma. Top 3 rows, hematoxylin and eosin staining; bottom row, CD31 immunohistochemistry staining.

**Figure 3:**
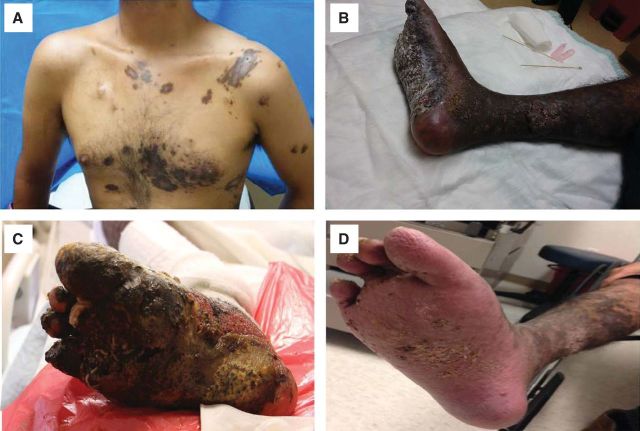
Skin lesions after the first dose of chemotherapy and (A, B) before, (C) during, or (D) 1 year after maggot debridement therapy.

Punch biopsies from the right lower extremity and left upper chest confirmed the diagnosis of Kaposi's sarcoma (Fig 2). A multidisciplinary team initiated a conservative approach to salvage his lower leg beginning with liposomal doxorubicin 20 mg/m^2^ once every 3 weeks to reduce underlying tumor (a total of 17 doses was given eventually). As the tumor burden improved on his face and torso (Fig 3A), a continuing obstacle to the patient's progress (and ability to walk) was the large circumferential tumor on the patient's right lower extremity (Fig 3B). Magnetic resonance imaging of right lower extremity 13 days after the first dose of liposomal doxorubicin showed extensive cellulitis and myositis of the foot with subcutaneous emphysema (possibly necrotizing) but no osteomyelitis, no communication of the plantar necrotic tissue and deeper viable tissue. Enzymatic debridement was determined to be of no benefit. Treating the patient with chemotherapy in the presence of this open, infected wound was considered extremely risky. Despite one chemotherapy treatment with liposomal doxorubicin, there was no improvement noted in the wound. It was felt that there was likely poor chemotherapy penetration in the highly necrotic tumor tissue. At this point the patient was facing potential death secondary to infection or an amputation of the involved extremity.

The decision was made to attempt two rounds of biologic debridement with MDT (Fig 3C) before using amputation as a last resort. The two treatments of MDT were undertaken at 20 and 27 days after the initial dose of liposomal doxorubicin. For each round, one vial of medical maggots (Monarch Labs, Irvine, CA) was applied and removed after 48 hours by a wound nurse, according to the protocol provided by the manufacturer and Maggot Debridement Therapy Protocol at our institute (Protocol No. 46421 MT). In detail, DuoDERM dressing was applied around the wound and maggots were placed directly onto the wound at a concentration of 5 to 10 per square centimeter of wound base. The entire area was reinforced with moist 4 × 4 gauze squares and wrapped in Kerlix. The dressing was assessed every 6 hours by a bedside nurse and the gauze dressing on top of the maggots was kept moistened with normal saline. Forty-eight hours later, the dressing and maggot cage dressing were removed with saline-soaked gauze, and then the entire foot was irrigated with sterile normal saline. Dead tissue was removed from the plantar area of the foot with additional sterile saline washes. Red tissue was noted at the plantar aspect of the foot and at the base of the great toe. The foot was then placed in a basin with sterile saline to soak for 1 hour before being dressed with oil-emulsion/Vaseline from right ankle to midcalf in dry gauze and Kerlix wrap. During the treatment, the patient reported that his pain level was average at a level of 2 on a scale of zero to 10, which was tolerable.

MDT profoundly decreased the necrotic burden surrounding the foot, allowing the viable tissue to arise. Repeat magnetic resonance imaging 3 days after the second round of MDT (also 11 days after the second dose of liposomal doxorubicin) showed decrease in myositis and cellulitis and stable cutaneous lesions. The patient was discharged home shortly after and continued to be observed through our multispecialty clinics. He continued with his chemotherapy regimen with continued response to his systemic lesions. Eight months later, the lesions on the patient's torso have melted away to less than 5% of their original presentation. The large lemon-size mass along the medial aspect of his right foot had completely resolved, and necrotic tissue on the plantar surface of the foot was replaced with viable tissue with intact sensation. One year later, his right leg remained intact (Fig 3D) and he was able to walk on his foot, although some of the skin changes persisted.

## DISCUSSION

Although MDT as a means of biologic debridement has been recognized for hundreds of year, its use is making a comeback in recent literature. Its use is well documented in diabetic foot ulcers and chronic nonhealing wounds, although use in malignancy-associated wounds has not been reported that often (Table 1). An extensive review of literature did not reveal any other case of successful MDT in a patient with Kaposi's sarcoma. It adds to the arsenal of tools available to control secondary infection and necrosis and promote healing in advanced malignant wounds of the skin.

**Table 1:** Literature Review of MDT on Cancer Wounds

Reference	Year of Publication	Patient		Primary Cancer	Site of Wound Where MDT Was Used	Response to MDT
		Sex	Age (years)			
Schouten et al^7^	2009	M	87	Basosquamous carcinoma of the right ear	Right ear	Spontaneous myiasis in the affected ear; upon removal, the wound looked cleaner and was absent of foul odors, and the carcinoma was subsequently removed surgically
Preuss et al^11^	2004	F	73	Squamous cell carcinoma of tongue base with bilateral metastases to the neck	Neck	Necrotizing fasciitis resolved within 4 days; red granulation tissue appeared at the surface, and the wound healed 6 weeks later because of secondary healing
Sealby et al^9^	2004	F	65	Endometrial adenocarcinoma with localized right foot metastases	Right foot	Wound started to heal; multiple rounds of MDT were used
Dunford et al^10^	2001	M	64	Mantle-cell lymphoma	Right thigh/groin	MDT was unsuccessful because the maggots traveled into the dressing and the wound had excessive exudate and lymph; complete wound healing in 14 weeks by multiple therapies, including vacuum-assisted closure and *Leptospermum* honey
Mumcuoglu et al^12^	1999	M	Not specified	Basal cell carcinoma	Left thumb	Specifics not provided
Jones et al^8^	1998	M	80	Squamous cell lung cancer	Chest	MDT cleared the infection and odors coming from lesion, but therapy was stopped because of the pain
Reames et al^5^	1988	M	60	Squamous cell carcinoma of the lower lip and neck	Neck	Spontaneous myiasis cleaned the wound with healthy granulation tissue; the wound became necrotic with a foul-smelling odor after the removal of maggots
Bunkis et al^6^	1985	F	88	Squamous cell carcinoma of the head	Right upper cheek	Spontaneous myiasis was kept on the wound and monitored for 3 days before removal; one week later, the wound was free of necrotic debris

Abbreviations: F, female; M, male; MDT, maggot debridement therapy.

Managing malignant wounds is a challenge because the underlying neoplastic process continues to demonstrate aberrant growth and necrosis, and the immunity and the healing capability of the host are often severely compromised by both the malignancy and chemotherapy. It is necessary to debride the wound to remove the foreign debris and devitalized or contaminated tissues from a wound bed so that the surrounding healthy tissues are exposed. Clinicians may debride wounds by using various methods, including surgery; conservative sharp, high-pressure fluid irrigation; ultrasonic mist; autolysis; or enzymatic agents. Among these, surgery is often the most effective option, but it also poses the most significant risk (in the case of our patient, it would have meant an above-the-knee amputation or death as a result of severe infection). Patients may not tolerate the surgical procedure or the wounds may heal poorly as a result of ongoing chemotherapy treatments.

MDT provides a natural, effective, cheap, and safe alternative for wound debridement. Successful wound debridement has been observed with naturally occurring myiasis.^6,7^ The first controlled clinical trials with MDT were not begun until 1990,^13^ and it was not until 2004 that the US Food and Drug Administration first granted marketing clearance to medicinal maggots (medical maggots; Monarch Labs) as a medical device (because of the physical action of the maggot over the wound).^14^ Maggots exclusively feed on (debride) only the dead (necrotic or gangrenous) tissue, hence leaving the viable tissue intact, which is a definite advantage over conventional surgical debridement. Besides, other beneficial effects on the wounds like microbial killing (disinfection) and hastened wound healing (growth stimulation) were also observed and have been confirmed by numerous clinical and laboratory studies.^15^

Scientific evidence regarding the mechanism of the three actions of MDT (debridement, disinfection, and growth stimulation) has accumulated slowly. The maggots do not bite off pieces of tissue. Instead, they secrete and excrete their digestive enzymes (alimentary secretions and excretions, including a wide array of matrix metalloproteinases and deoxyribonuclease, and so on) to liquefy the necrotic tissue, and the maggots can then easily imbibe it.^15^ The physical movement of the maggots over the wound, plowing the tissue and spreading their alimentary secretions and excretions as they go, contributes significantly to the debridement effort. The maggots disinfect the wound by ingesting bacteria, releasing antimicrobial peptides, and scraping away and dissolving biofilm. Deoxyribonuclease plays an important role not only in debridement but also in inhibiting microbial growth and biofilm. In both in vitro and in vivo^16^ studies, MDT was found to be more effective in wounds involving Gram-positive bacteria. The disinfecting capability of maggots is particularly valuable given the rising incidence of antibiotic resistance. Growth stimulation and wound healing are promoted by production of allantoin and urea, fibroblast proliferation and migration, angiogenesis, increased perfusion, reduced inflammatory response, and the release of growth factors. It has been demonstrated that maggot-treated wounds follow normal wound healing phases.^17^

Maggot debridement therapy is generally safe. Current standard MDT uses freshly emerged and sterile larvae of the common green bottle fly, *Phaenicia (Lucilia) sericata*, which is a type of artificially induced myiasis raised under controlled clinical conditions, thus minimizing the risk of undesired infection by the bacterial population of the insect's intestinal tract and integument. The most common complication associated with MDT is pain. A retrospective analysis of 41 patients^18^ reported that 40% of nondiabetic patients experienced more pain during MDT than before, although diabetic patients experienced the same amount of pain before and during the therapy. In 78% of patients, pain can be adequately treated with analgesics. Another potential severe complication is bleeding; however, only one case report of serious bleeding in an 87-year-old woman treated for a mixed arterial-venous ulcer of the right leg has been reported so far.^19^

In our patient case, MDT helped debride, disinfect, and heal the Kaposi's sarcoma wound, avoided an above-the-knee amputation and potential death as a result of severe infection, and allowed time for the chemotherapy and anti-HIV medications to be effective in the patient. It has been reported that patients with chronic limb ischemia are less likely to benefit from MDT,^3^ because good blood circulation is essential for wound healing. We hypothesize that malignant wounds are good candidates for MDT because tumors are associated with neovascularization leading to areas of high vascularity in and around the tumor. Even if wound closure (healing) is not always achieved, MDT can help control infection, reduce odor, and avoid possibly deforming surgeries.^20^ Thus, MDT is a promising, cost-effective (US$150 for each vial containing 250 to 500 disinfected maggots; one vial was used at each treatment in our patient), and worth-a-try modality in the management of malignant wounds, especially in the areas of the world and in settings with limited health care resources. Larger, well-designed, randomized studies are needed to further elucidate the feasibility, technique standardization, effectiveness, and potential complications of MDT in malignancy wounds.
